# Identification and Characterization of a New Type of Holin-Endolysin Lysis Cassette in *Acidovorax oryzae* Phage AP1

**DOI:** 10.3390/v14020167

**Published:** 2022-01-18

**Authors:** Muchen Zhang, Yanli Wang, Jie Chen, Xianxian Hong, Xinyan Xu, Zhifeng Wu, Temoor Ahmed, Belinda Loh, Sebastian Leptihn, Sabry Hassan, Mohamed M. Hassan, Guochang Sun, Bin Li

**Affiliations:** 1 State Key Laboratory of Rice Biology and Ministry of Agriculture Key Laboratory of Molecular Biology of Crop Pathogens and Insects, Institute of Biotechnology, Zhejiang University, Hangzhou 310058, China; 11816060@zju.edu.cn (M.Z.); 21616122@zju.edu.cn (J.C.); 21816076@zju.edu.cn (X.H.); 12016074@zju.edu.cn (X.X.); 21916082@zju.edu.cn (Z.W.); temoorahmed@zju.edu.cn (T.A.); 2 State Key Laboratory for Managing Biotic and Chemical Threats to the Quality and Safety of Agro-Products, Zhejiang Academy of Agricultural Sciences, Hangzhou 310021, China; ylwang88@aliyun.com; 3 Zhejiang University-University of Edinburgh Institute, Zhejiang University, Hangzhou 314400, China; belinda.loh@intl.zju.edu.cn (B.L.); leptihn@intl.zju.edu.cn (S.L.); 4 Department of Biology, College of Science, Taif University, Taif 21944, Saudi Arabia; Hassan@tu.edu.sa (S.H.); m.khyate@tu.edu.sa (M.M.H.)

**Keywords:** *Acidovorax oryzae* phage AP1, lysis cassette, holin, endolysin

## Abstract

Phages utilize lysis systems to allow the release of newly assembled viral particles that kill the bacterial host. This is also the case for phage AP1, which infects the rice pathogen *Acidovorax oryzae*. However, how lysis occurs on a molecular level is currently unknown. We performed in silico bioinformatics analyses, which indicated that the lysis cassette contains a holin (HolAP) and endolysin (LysAP), which are encoded by two adjacent genes. Recombinant expression of LysAP caused *Escherichia coli* lysis, while HolAP arrested growth. Co-expression of both proteins resulted in enhanced lysis activity compared to the individual proteins alone. Interestingly, LysAP contains a C-terminal region transmembrane domain, which is different from most known endolysins where a N-terminal hydrophobic region is found, with the potential to insert into the membrane. We show that the C-terminal transmembrane domain is crucial for protein localization and bacterial lysis in phage AP1. Our study characterizes the new phage lysis cassette and the mechanism to induce cell disruption, giving new insight in the understanding of phage life cycles.

## 1. Introduction

Phages employ two different strategies to release progeny phage from a host bacterial cell. Some simple ssDNA or RNA phages of Gram-negative bacteria rely on a single gene to lyse the bacteria by inhibiting the synthesis of peptidoglycan in the bacterial cell wall [1]. Most dsDNA tailed phages use efficient and specific “holin-endolysin” two-component lysis cassettes to induce lysis in the host cells [2]. The endolysin, which is a peptidoglycan-degrading enzyme, accumulates in the cytosol at the end of the replication cycle [3,4,5]. The holin, a small hydrophobic membrane spanning protein, is essential for the endolysin to translocate across the membrane to enter the periplasm [6]. Holins form membrane lesions in the cytoplasmic membrane at a genetically predetermined time, which permeabilizes the inner membrane allowing the endolysin to cross this barrier [7]. The cell will then burst due to the degradation of the peptidoglycan, allowing the mature phage particles to be released [8].

Interestingly, endolysins are also exported via a holin-independent mechanism. In this case, holin does not form macropores on the membrane to release endolysin, but activates the release of endolysin to the periplasmic to lyse the cell wall by dispersing the proton dynamic potential, so as to control the time of lysis. However, there is a difference between Gram-positive and Gram-negative bacterial phages in holin-independent mechanism. For example, endolysin Lys44 of Gram-positive bacterium *Oenococcus oeni* phage fOg44 was found to contain a cleavable signal peptide, which hijacks the host–transport system to facilitate its translocation into the periplasm [9] However, endolysins are exported in Gram-negative bacteria by connecting with signal-arrest-release (SAR) sequences [10]. For instance, the endolysins of *Siphoviridae* bacteriophage swi2 with hydrophobic and positively charged amino acids at the N-terminus showed strong activity to naturally lyse Gram-negative bacteria [11]. The coliphages P1 and 21 encode the proteins Lyzp1 and R21, which are functional peptidoglycan-degrading enzymes, yet do not have typical cleavable signal peptides but a SAR region in the N-terminal region, which serves as a signal-arrest domain that facilitates the secretion of the endolysin via the Sec translocon [12,13].

In this study we systematically investigated the lysis cassette of phage AP1 using a variety of approaches. We found that LysAP is different from other known endolysins as it does not encode the traditional SAR N-terminal transmembrane domain (TMD), but employs the same strategy: the secretion across the membrane through the Sec pathway.

## 2. Materials and Methods

### 2.1. Bacterial Strains and Growth Conditions

The bacterial strains and plasmids used in this study are indicated in Table 1. Ao strains were cultured in Luria–Bertani (LB) agar or broth medium (Oxoid Ltd., Hampshire, UK) at 30 °C [14]. *E. coli* strains were grown in LB agar or broth medium at 37 °C. If necessary, kanamycin (50 μg/mL), ampicillin (100 μg/mL), or IPTG (isopropyl-β-d-thiogalactopyranoside; 1 mM) was added to the medium (Sangon Biotech Co., Ltd., Shanghai, China).

### 2.2. Bioinformatics Analysis

DNAMAN v. 6 and SnapGene Viewer v. 2.2 software were used to analyze the position, base composition, and GC content of HolAP and LysAP coding genes; NEBcutter (http://nc2.neb.com/NEBcutter2/, accessed on: 10 January 2021) Prediction of restriction enzyme and other information. Application of ExPASY prot param tool (http://www.expasy.org/proteomics/protein, accessed on: 15 March 2019) to predict the physicochemical properties of HolAP and LysAP proteins, including isoelectric point, molecular weight, stability index, aliphatic index, hydrophobicity, hydrophilicity, and enzyme digestion characteristics. Applying blastp in NCBI (https://blast.ncbi.nlm.nih.gov/Blast.cgi, accessed on: 7 June 2021) to carry out the conservative functional domain analysis of phage AP1 HolAP and LysAP. TMHMM Server v. 2.0 (http://www.cbs.dtu.dk/services/TMHMM/, accessed on: 15 March 2019), I-TASSER (https://zhanglab.ccmb.med.umich.edu/I-TASSER/, accessed on: 15 March 2019), Signal P 4.0 Server (http://www.cbs.dtu.dk/services/SignalP-4.0/, accessed on: 15 March 2019) were applied to analysis the biological characteristics and secondary structure of HolAP and LysAP proteins, such as transmembrane domain, signal peptide and coiled coil. The 3D structure of LysAP protein was predicted through the website (https://zhanglab.ccmb.med.umich.edu/I-TASSER/, accessed on: 15 March 2019) using parameters with default values. Protein active sites were predicted according to the references [12,13]. Genomes of *Acidovorax* and other related bacterial genus phages were downloaded from NCBI. DNAMAN v. 6 was used to align the sequence to show the conservatism of LysAP.

### 2.3. Standard DNA Manipulation, PCR and DNA Sequencing

Genomic DNA of phage AP1 was extracted using traditional phage genome extraction ways with slight modification [16]. In brief, phage lysates were centrifuged at 11,000× *g* for 15 min at 4 °C to remove cell debris. Then, the supernatants were concentrated and genomic DNA was extracted using phage genome extraction kit (Sangon Biotech Co., Ltd., Shanghai, China). Procedures for the isolation of plasmid DNA, DNA amplification by PCR, PCR product purification and DNA sequencing were performed according to standard procedures [16] or in accordance with the manufacturer’s protocol (Axygen, Tewksbury, MA, USA). All other enzymes were purchased from Thermo.

### 2.4. Recombinant Plasmids Construction

The DNA inserts for these constructs were PCR-amplified from the following sources: for pETDuet-HolAP, pET-28a-HolAP, and pCH-HolAP, the HolAP gene was from AP1 DNA; pETDuet-LysAP, pET-28a-LysAP_ΔTMD_, and pKNT-LysAP, the LysAP gene was from AP1 DNA; for pETDuet-HolAP-LysAP, the HolAP and LysAP gene was from AP1 DNA; the PCR product was digested with and cloned into unique NcoI and BamHI restriction sites in the ampicillin resistance plasmid pETDuet-HolAP; the PCR product was digested with and cloned into unique NdeI and BglII restriction sites in the ampicillin resistance plasmid pETDuet-LysAP; the PCR product was digested with and cloned into unique HindIII and EcoRI restriction sites in plasmid pCH-HolAP; and the PCR product was digested with and cloned into unique BamHI and EcoRI restriction sites in plasmid pKNT-LysAP. The plasmid pET-28a-LysAP_ΔTMD_ in which the transmembrane domain (TMD) was knocked out, was under control of the lac promoter. All the primers used in this study were listed in Table 2.

### 2.5. Growth Measurement

Bacterial growth was determined by measuring the OD600 values using Microplate Spectrophotometer (Thermo Fisher Scientific Inc., Waltham, MA, USA) [17]. In brief, 50 μL of freshly grown overnight culture was used to inoculate 5 mL, at OD600 = 0.6, induction was initiated by adding IPTG (1 mmol/L) and incubating at 37 °C, 200 rpm. LB broth without bacteria was used as the negative control. The experiment was repeated three times with three replicates of each treatment. To block the SecA secretory system, NaN_3_ (1 to 10 mM) was added simultaneously with induction [18].

### 2.6. Protein Expression, Purification and Western-Blotting

An overnight culture of *E. coli* BL21(DE3) harboring recombinant plasmid were diluted 1:100 into 250 mL of LB medium and incubated at 37 °C and 200 rpm. At an OD600 of 0.6, production of proteins was induced by the addition of IPTG to 1mM. After incubation for 4–8 h at 30 °C and 200 rpm, the cells were harvested, and the pellet was resuspended in 20 mL of native lysis buffer (300 mM NaCl, 50 mM NaH_2_PO_4_, 10 mM imidazole; pH 8.0). The cells were lysed using Ultrasonic processor (SXSONIC, Shanghai, China). The protein was purified by ProteinIso^®^ Ni-NTA Resin (TransGen Biotech, Beijing, China) following the manufacturer’s protocol. Proteins were applied to sodium dodecyl sulfate (SDS)-polyacrylamide gels, and the separated proteins were stained with Coomassie blue. Western blotting was conducted using Anti-His tagged antibody following the reported protocols [16].

### 2.7. Detection of β-Galactosidase Activity

β-galactosidase activity was conducted following the previous report with modification [2]. A volume of 50 μL of overnight bacterial culture was used to inoculate 5 mL LB broth and cultured at 37 °C, 200 rpm. When OD600 reached 0.4, IPTG was added and induction was conducted at 20 °C for 6 h, 12 h, and 24 h. The culture was centrifuged at 12,000 rpm for 5 min, after which a 500 μL aliquot of extracellular supernatant was added to 100 μL of ortho-Nitrophenyl-β-galactoside (ONPG) (20 mM). The mixture was incubated in a 45 °C water bath for 30 min. To stop the reaction, 600 μL Na_2_CO_3_ (0.5 mM) was added. The β-galactosidase activity was determined by measuring the optical density at 420 nm (OD420) using a microplate photometer.

### 2.8. Live/Dead Cell Staining

Bacterial lysis was determined by live/dead cell staining [19]. Briefly, 5 mL of LB broth was inoculated with 50 μL of overnight culture and cultured at 37 °C, 200 rpm. At OD600 = 0.6, induction was initiated with IPTG and incubated at 37 °C for 30 to 60 min. Live/dead staining assay was conducted with the BacLight bacterial viability kit (Invitrogen). The kit includes two nucleic acid stains, a red-fluorescent (propidium iodide stain, PI) for dead bacteria, and a green fluorescent (SYTO 9 stain) for live bacteria. Fluorescence was detected using an inverted confocal microscope (Leica-SP8, Heidelberg, Germany).

### 2.9. Microscopy Analysis

Bacterial sample preparation for TEM was conducted as previously described with some revision [20]. Briefly, bacteria were collected by centrifugation at 5000 g for 5 min, then washed 3 times with 0.1 M PBS solution followed by fixing with 2.5% (*v/v*) glutaraldehyde. The samples were then stained with 1% (*w/v*) osmium tetroxide in 0.1 M PBS for 1 h at room temperature, then washed three times with 0.1 M PBS. Following this, the samples were dehydrated stepwise over a range of ethanol solutions (70%, 80%, 90%, 95%, and 100% *v/v*) with each step lasting for 15 min at room temperature. Dehydrated samples were embedded in Epon 812, a low-viscosity embedding resin. TEM (JEM-1230, JEOL, Akishima, Japan) was used to observe the changes in bacteria according to the operating methods. For Gram staining, bacterial strains to be observed were collected and washed by PBS (pH 7.2) twice. Gram Stain Kit (Solarbio, Beijing, China) was used and the stained bacteria were observed through microscopic examination.

### 2.10. Bacterial Two-Hybrid Assays

Bacterial two-hybrid assays were performed similarly to what was described previously [21]. The coding region of LysAP (excluding the stop codon) was amplified by PCR using primers pKNT-LysF and pKNT-LysR. The PCR products were digested with *Bam*HI and *Eco*RI and were cloned into the plasmid pKNT25, resulting in pKNT-LysAP. Similarly, the PCR product of HolAP was digested with *Hind*III and *Eco*RI and cloned into the same sites of pCH363, generating pCH-HolAP. Positive and negative controls were stored by lab. BTH101 expressing the motA and ypfA proteins was used as the positive control, while BTH101 with no plasmids was used as the negative control [15].

To introduce recombinant plasmids into the *E. coli* host strain BTH101, 5 μL of each pair of the recombinant plasmids was mixed with 100 μL of chemically competent cells of BTH101. Samples were incubated at 4 °C for 30 min and then heat shocked at 42 °C for 90 s. An 800 μL volume of LB broth was added to the heat-shocked cells, and cells were incubated with shaking for 1 h at 37 °C. Cells were concentrated and spread on LB plates supplemented kanamycin (50 μg/mL) and ampicillin (100 μg/mL). Plates were incubated overnight at 37 °C. Single colonies were picked and grown at 37 °C in LB broth plus kanamycin (50 μg/mL) and ampicillin (100 μg/mL) with vigorous shaking. Then, 5 μL aliquots of cells (optical density at 600 nm (OD600), 1.0) were spotted on LB plates supplemented with 40 μg/mL 5-bromo-4-chloro-3-indolyl-β-d-galactopyranoside (X-Gal), 500 μM IPTG, 100 μg/mL ampicillin, and 50 μg/mL kanamycin. Plates were incubated for 48 h at 23 °C before imaging.

## 3. Results

### 3.1. In Silico Description of AP1 Lysis Cassette

**Figure 1:**
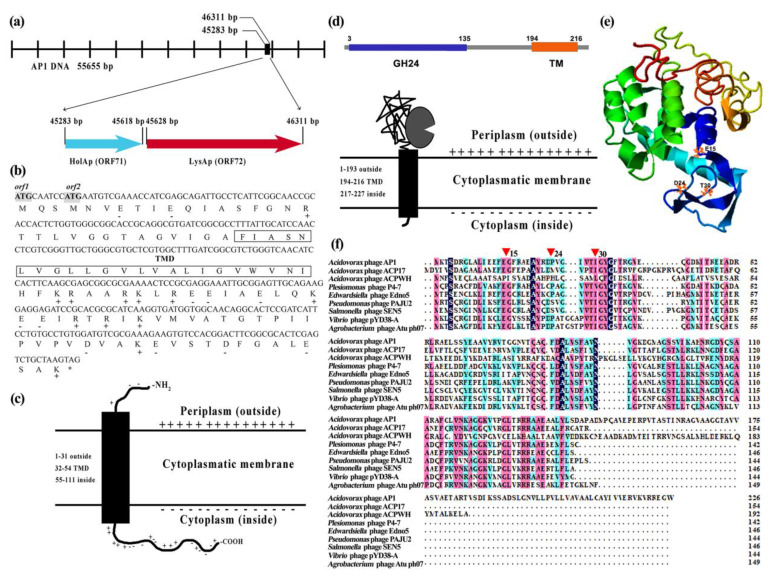
In silico characterization of the AP1 lysis cassette: (**a**) Genomic organization of HolAP and LysAP in the genome of phage AP1. (**b**) Analysis of amino acids in HolAP. Charged residues are indicated by a + or − sign; The transmembrane domain (TMD) is indicated with a box; Potential translation start codons are highlighted in grey. (**c**) Topological model of HolAP. (**d**) Domain organization (top) and model of LysAP (bottom) with the hydrolase domain indicated by the “Pac-Man” shape. (**e**) 3D structure prediction of LysAP. (**f**) Sequence alignment of endolysin LysAP with that of *Acidovorax* phage ACP17 (YP_009609701.1), *Acidovorax* phage ACPWH (AXY83360.1), *Plesiomonas* phage P4-7 (ANW09608.1), *Edwardsiella* phage Edno5 (AYP69211.1), *Pseudomonas* phage PAJU2 (YP_002284361.1), *Salmonella* phage SEN5 (YP_009191752.1), *Vibrio* phage pYD38-A (YP_008126192.1), and *Agrobacterium* phage Atu_ph07 (ASV44718.1). Red triangles (E15, D24, T30) represent the catalytic triad residues.

We first analyzed the phage genome (GenBank accession number OM049504) and lysis cassette of phage AP1 using bioinformatics. The AP1 genome region encoding the lysis cassette contains two open reading frames, HolAP (ORF71) and LysAP (ORF72), which encode a putative holin and an endolysin, respectively (Figure 1a). The lysis genes of AP1 appear to be arranged in canonical order, such that HolAP (45,283–45,618 bp) is located upstream of LysAP (45,628–46,311 bp), which is consistent with the genetic architecture of lysis cassettes found in most phages infecting Gram-negative bacteria.

HolAP is a small protein, predicted to be composed of 111 amino acids with a molecular weight of 11.9 kDa (Figure 1b). Protein sequence analysis shows that HolAP belongs to the phage holin 2_3 superfamily. HolAP has a type III holin structure: a membrane protein with a transmembrane region (amino acids 32–54), the N-terminal region of which is found in the periplasm while the C-terminus of the protein is located in the cytoplasm and is rich in positively and negatively charged amino acids (Figure 1c).

LysAP is larger with 227 amino acids and a molecular weight of 24.5 kDa. Bioinformatic analysis indicates that there is no typical signal peptide, and conserved domain analysis showed that the presence of two regions with a lysozyme-like domain, a member of GH24 family, which is thought to display glycosyl hydrolase activity (amino acids 3–135) and a transmembrane domain (TM; 194–216 amino acid sites) (Figure 1d). GH24 shows the conserved catalytic triad (E15, D24, and T30) (Figure 1e), similar to many phage lysozymes (Figure 1f). Notably, the C-terminal of LysAP was relatively unique although having high sequence homology to other phages. A SAR peptide is located in the C-terminal region, which is in stark contrast to the known, “traditional” endolysin topology (N-terminal).

### 3.2. Holin HolAP Inserts in the Cell Membrane and Interacts with Endolysin LysAP

**Figure 2:**
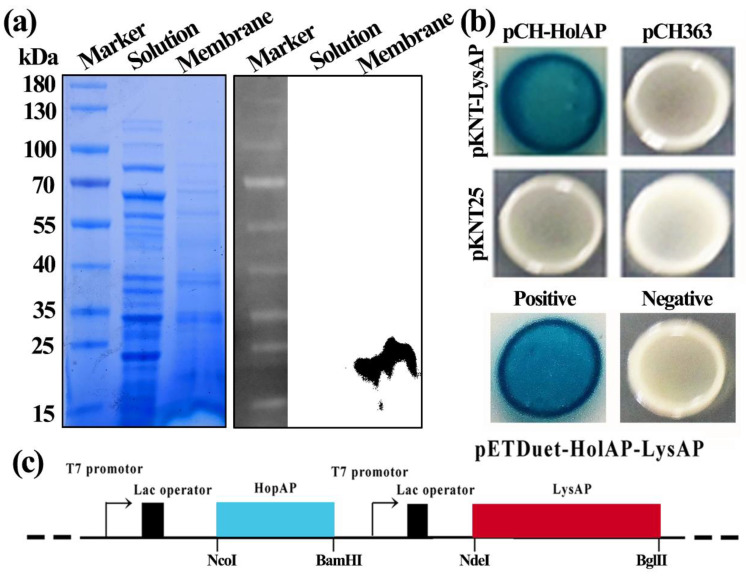
(**a**) Sub-cellular localization of protein HolAP by coomassie gel (**left**) and Western blotting assay (**right**). Anti-His antibody was used to detect for target proteins. (**b**) Protein interaction identified between HolAP and LysAP by bacterial-2-hybrid. (**c**) Construction of co-expression plasmid pETDuet-HolAP-LysAP.

pET-type plasmids have been widely used to express bacteriophage-derived lytic enzymes [22], such as holins and endolysins [23,24], which provides us with a vector to study the proteins in vitro and their activity in cells. Thus, in this study, we cloned the respective proteins into *E. coli* expression vectors (pET-28a) and introduced the plasmids into *E. coli* strain BL21 (DE3). Following IPTG induction, intracellular and membrane protein samples were collected from the induced *E. coli* BL21 cells expressing pET-28a-HolAP. Furthermore, Western blotting analysis indicated no protein band was observed in the cytoplasmic fraction, in contrast, a single band with a molecular weight of about 17 kDa was detected in isolated membranes. This result confirmed that HolAP is a membrane protein (Figure 2a).

In many lysis systems, holins facilitate the translocation of endolysins into the periplasm. However, it is unclear if the proteins interact with each other since holins could form non-specific pores that allow the leakage of cytoplasmic content out of the cell. We therefore employed bacterial two-hybrid to investigate if HolAP and LysAP interact. In this approach, a direct interaction of the two proteins allows the association of two fragments of an enzyme that ultimately leads to the production of β-galactosidase, which can be detected by cleavage of X-Gal forming blue-colored colonies. When expressing HolAP together with LysAP, we obtained the same result as that observed for the positive control, indicating a direct interaction (Figure 2b).

### 3.3. Expression of AP1 Lysis Cassette Leads to Cell Lysis

**Figure 3:**
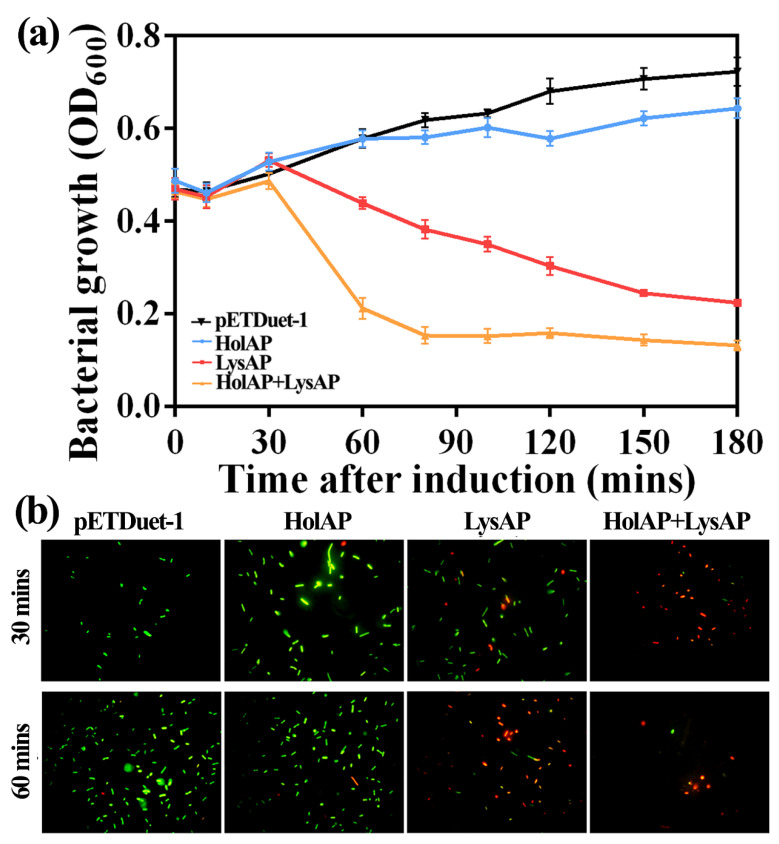
Effects of AP1 lysis cassette protein expression on bacterial growth: (**a**) Growth curves of bacteria (**b**) Fluorescent staining using a live-dead stain (live: green, dead: red).

We constructed three plasmids to test the impact of protein expression in *E. coli* BL21 (DE3): Two plasmids encoded HolAP and LysAP separately while a third plasmid allowed the co-expression of both proteins (HolAP-LysAP) together. The expression of HolAP alone, similar to the negative control (pETDuet-1), had no impact on bacterial growth, assessed by the absorbance at 600 nm (OD600). However, when protein expression was induced in cells containing the co-expression plasmid, cell lysis occurred 30 min post induction, with the solution becoming viscous displaying cell debris typical for phage-induced lysis. The observed decrease was larger than that of LysAP alone, which also showed a reduction in growth (Figure 3a), indicating that LysAP is able to induce growth arrest or lysis; however, not as effectively as in concert with HolAP.

We then performed a live–dead stain to assess whether the cells expressing the proteins are indeed lysed or only arrested in growth, or if the observed decrease in light absorbance is due to other factors such as morphological changes. *E. coli* cells expressing HolAP alone showed green fluorescence, an indicator that the bacteria are alive, which is consistent with our observation of the growth curves. In contrast, a high ratio of cells expressing LysAP were stained red, indicating that the expression of the protein leads to the collapse of the membrane integrity, thus killing the cells. The ratio of dead to live cells increased over the time as more protein was expressed. However, most of the *E. coli* cells co-expressing HolAP and LysAP proteins together were already stained red after a brief induction time of 30 min, indicating that most of the bacteria died (Figure 3b), with even more pronounced effects after 1 h. These findings suggest that the expression of HoLAP alone does not cause bacterial death. Expression of LysAP alone causes cell death, however to a lesser extent than that caused by the co-expression of HolAP and LysAP together. Therefore, HolAP is not responsible for cell lysis, but accelerates the process significantly, demonstrating the concerted action of the protein together with the SAR endolysin.

### 3.4. Expression of AP1 Lysis Cassette Proteins Affect Membrane Integrity and Induce Morphological Changes

**Figure 4:**
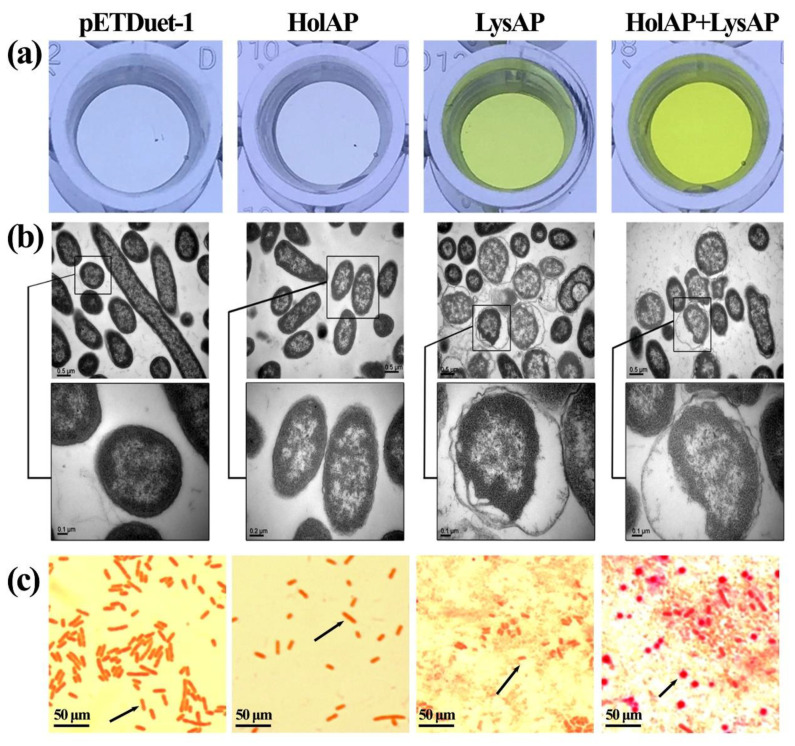
Effects of AP1 lysis cassette expression on cell membrane permeability: (**a**) Detection of β-galactosidase. (**b**) Morphological changes in cells under TEM. (**c**) Gram-stained cells observed under optical microscope. The arrows show the morphological changes in bacteria.

As we had observed that the expression of LysAP and protein production of LysAP, together with HolAP, led to cell death in *E. coli*, we used an additional method to demonstrate that the integrity of the membrane was compromised. β-galactosidase is a cytosolic enzyme that cannot cross the membrane barrier unless the cell envelope exhibits defects larger than the protein. By using this approach, we determined the increase in absorbance of β-galactosidase in cells expressing either protein or both. As shown in Figure 4a, HolAP (*E. coli* expressing HolAP alone) had a similar color with the negative control pETDuet-1 (*E. coli* containing the empty vector) with the OD420 of 0.125 and 0.126, respectively, indicating that the membranes remained intact. However, the color of LysAP turned yellow with the OD420 of 0.368, which can be attributed to the fact that the expression of LysAP destabilizes bacterial membrane, resulting in cells lysis, and the release of the enzyme. This change in color was even more pronounced in cells expressing both LysAP and HolAP with the OD420 of 0.88, which was significantly higher than that of the HolAP and LysAP alone. This clearly shows that the concerted action of both proteins results in the destruction of the bacterial cell envelope, leading to the leakage of cell contents.

The morphological changes in *E. coli* BL21 (DE3) expressing genes in the lysis cassette were verified by transmission electron microscope (TEM) (Figure 4b). Consistent with the β-galactosidase activity assay, the morphology of cells serving as a negative control was normal, the structure of the cell wall and cell membrane were unaltered, the density of the cellular contents appeared high, and the cell color was dark. In comparison, the cell wall and cell membrane of *E. coli* expressing HolAP had shrunk slightly, the density of the cellular content had decreased and the color of the cell appeared much lighter. This may be due to the expression of HolAP protein, which may cause the change in membrane permeability by forming pores. In the case of *E. coli* cells expressing LysAP alone, or during the co-expression of HolAP with LysAP for 30 min, the bacterial cell membrane shrunk dramatically, the cell wall structure became irregular, and the cell color became light, indicating disintegration of the cellular envelope.

In addition, Gram-stained cells expressing HolAP alone did not have an impact on the shape of the bacteria; however, the bacteria became shorter and adhered to each other when LysAP was expressed. The cells adopted a spherical morphology prior to lysis. Moreover, when the two proteins were expressed together, the cell boundary became less clear, and the shape changed from rod-shaped to spherical, with bacteria adhering to each other, in addition to the complete disintegration of the cells and the formation of cell fragments (Figure 4c). The above findings indicate that HolAP cooperated with LysAP to mediate bacterial lysis while having an impact on the morphology of the bacteria.

### 3.5. The C-Terminal Transmembrane Domain (TMD) Plays an Important Role in LysAP Lysis

**Figure 5:**
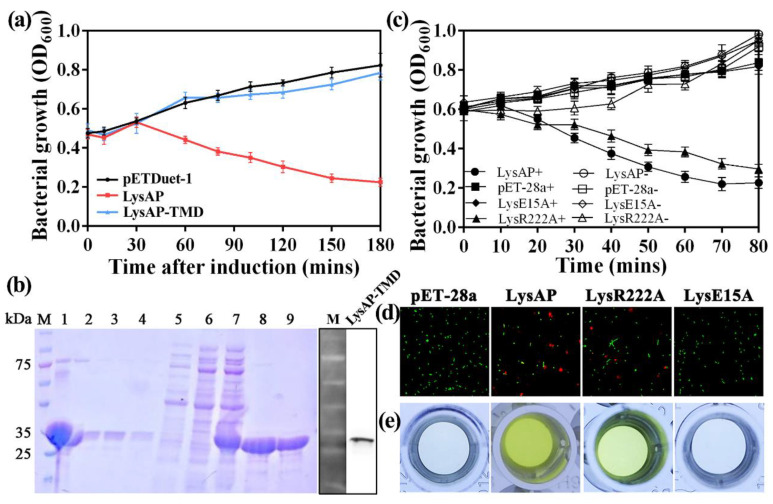
(**a**) Effect of LysAP_ΔTMD_ expression on bacteria growth. (**b**) Purification of LysAP_ΔTMD_. M, maker; 1, Cell lysate; 2–4, Flow through; 5, 6, Wash; 7–9, Elution. (**c**) Growth curve of *E. coli* BL21(DE3) carrying pET-28a-LysE15A and pET-28a-LysR222A. LysAP: BL21(DE3) carrying pET-28a-LysAP as positive control; pET-28a: BL21(DE3) carrying pET-28a as negative control; LysE15A and LysR222A: BL21(DE3) carrying pET-28a-LysE15A and pET-28a-LysR222A recombined plasmid respectively. “+” means adding 1 mM IPTG and “−” means not adding IPTG. (**d**) Staining of live or dead bacteria. (**e**) Detection of extracellular β-galactosidase activity.

Since the transmembrane domain is located in 194–216 amino acid sites of LysAP, we constructed LysAP_ΔTMD_ through deleting the 194–216 transmembrane domain and connecting the 193 amino acid with the 217 amino acid directly. Recombinant production of the truncated protein LysAP_ΔTMD_ appeared not to be toxic for the host cell, which is in contrast to the production of the full length endolysin LysAP. As described before, when LysAP was expressed alone, OD600 dropped by almost two thirds after three hours of induction. In contrast, in the presence of the TMD deletion mutant, the OD600 value increased indicating cell growth, exhibiting almost no difference compared to the negative control (Figure 5a). The large quantities of LysAP lacking the C-terminal transmembrane region in cells suggested that LysAP could not be anchored in the cell membrane, but accumulated in the cytosol after the C-terminal transmembrane region of LysAP was deleted (Figure 5b). These findings strengthen in silico predictions that the C-terminal TMD is involved in the transport of LysAP.

Analysis of the amino acids sequence of LysAP reveals that there are only four cationic amino acids R218, K220, R222, and R223 near the end of the TMD, which may affect the stability of TMD and the transport of LysAP. Representative point mutations (pET-28a-LysR222A and pET-28a-LysE15A) were constructed to explore the function of cationic amino acids and conserved catalytic triads. Growth ability assay showed that conserved amino acid site E15 is an important factor affecting LysAP lysis function while the cationic amino acid R222 has no significant effect on cell lysis. When pET-28a-LysR222A was inducted, the OD600 decreased continuously from the initial 0.6 to 0.3 within 80 min, and the number of bacteria decreased by 50%, which was consistent with the cleavage trend of the positive control (pET-28a+), which showed that R222A had a limited effect on LysAP lysis function. In comparison, the growth curves of pET-28a-LysE15A and the negative control were basically the same, both maintained the normal growth trend of bacteria, and their OD600 values gradually increased, reaching more than 0.7 after 80 min, indicating that E15A can effectively prevent LysAP from playing its cleavage function (Figure 5c).

This result was further verified through a staining test of live/dead bacteria (Figure 5d) and β-galactosidase activity assay (Figure 5e). Under the induction of IPTG for 30 min, *E. coli* carrying pET-28a-LysR222A plasmid were stained and observed. Results showed that most of the bacteria emitted green fluorescence while some of them were red (around 24%), indicating that some bacteria died, which was consistent with the LysAP expression (the bacterial death rate was 35%). *E. coli* carrying pET-28a-LysE15A plasmid and the negative control (pET-28a) showed nearly all fluoresced green, which indicated that almost all of the bacteria survived normally (Figure 5d). The results of the β-galactosidase activity test showed that the supernatant of the recombinant plasmid carrying pET-28a-LysE15A and the negative control was transparent with the OD420 of 0.131 and 0.123, respectively; however, the supernatant of *E. coli* carrying pET-28a-LysR222A and LysAP turned yellow with the OD420 of 0.283 and 0.359, respectively (Figure 5e). The above experiments also confirmed that the conserved active site E15 was an important site affecting the cleavage function of LysAP, while the effect of cationic amino acid R222 was not significant.

### 3.6. Sec System Is Involved in the Release Process of LysAP

**Figure 6:**
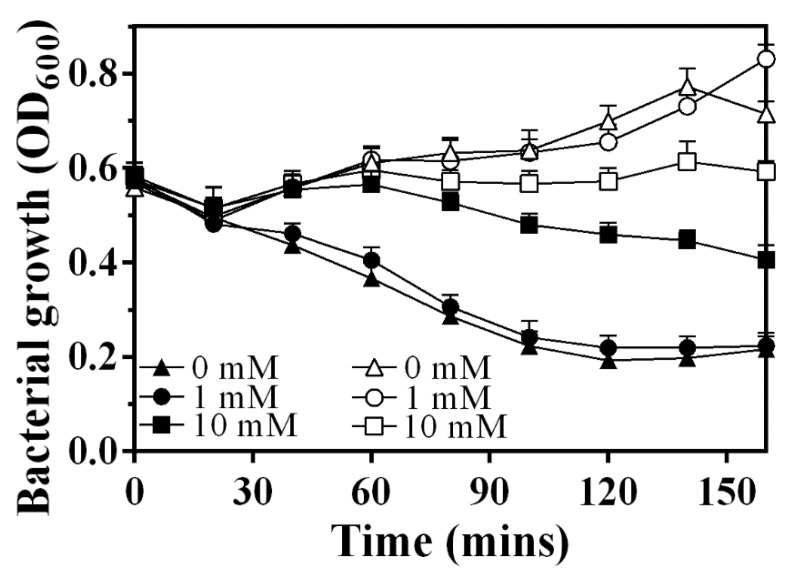
Growth curve of BL21(DE3) carrying pET-28a-LysAP recombinant plasmid. Solid and hollow icon means the conditions with or without IPTG induction, respectively; 0 mM, 1 mM, and 10 mM represent the addition of NaN_3_ with different concentrations.

Sodium azide (NaN_3_) has been widely used as an inhibitor of the ATPase activity of SecA, which is necessary for translocation of endolysin across the membrane [13,18,25]. Therefore, we used NaN_3_ to examine the involvement of the Sec system during LysAP production (Figure 6). Under the condition of adding 1 mM IPTG and 0 mM sodium azide, the OD600 of *E. coli* expressing LysAP decreased from 0.6 to about 0.2 within 160 min, which is similar to that when changing the NaN_3_ concentration to 1 mM, indicating that low concentrations of NaN_3_ could not effectively prevent lysis. Moderately increasing the concentration of NaN_3_ can effectively postpone lysis of the expression culture further (10 mM), which is consistent with the reported results. These observations suggest the involvement of the Sec machinery in the secretion of LysAP to the periplasm.

### 3.7. Lysis Model for C-Terminal SAR Endolysin-Holin Cassette

**Figure 7:**
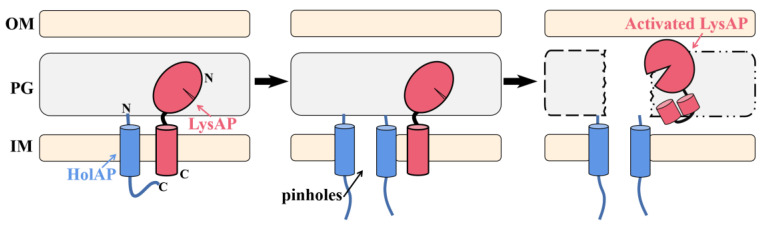
Lysis model for C-terminal SAR endolysin-holin cassette: HolAP and LysAP are anchored to the cell membrane (**left**), then HolAP formed pinholes (**middle**), and LysAP was activated and released (**right**). OM: Outer membrane; PG: Peptidoglycan; IM: Inner membrane.

The Sec-dependent signal sequence and its essential nature for significant enzymatic activity confirm that LysAP is a C-terminal SAR endolysin. In addition, the observation of a stronger lysis effect in the expression culture after co-expression of HolAP-LysAP is consistent with the role of HolAP as a pinholin. Based on the experimental results, a probable lysis model for C-terminal SAR endolysin–holin cassette is proposed (Figure 7). HolAP and LysAP are anchored to the cell membrane, pinholes are formed afterwards, and LysAP is activated and released due to pinholin-induced membrane depolarization, which eventually leads to bacterial cell lysis.

## 4. Discussion

The lysis cassette in most phages of Gram-negative hosts has been shown to require holin and endolysin, which aims at cytoplasmic membrane and peptidoglycan, respectively [2,26,27]. A third class of lysis proteins, the spanins, which attack outer membrane, was also included in some of the lysis cassettes [28]. In this study, a new type of holin–endolysin lysis cassette in *A. oryzae* phage AP1 was identified and no putative spanin was found, which was consistent with the result of Holt et al. [29], who found that ~15% of phages lack a spanin gene through bioinformatics analysis.

Holin is a small membrane protein produced by many phages at the end of the lytic cycle [6,30,31]. There are two main functions of holins in the phage lysis system. One is to form holes in the cell membrane (allowing 500 kDa protein to pass through) and release endolysin without signal peptide. These holes are nonspecific and allow endolysin and other proteins to pass through [8,32]. Holin can also act as a timer to regulate the phage cracking of bacteria precisely [33,34]. In this study, through bioinformatics analysis, we predicted that there was only one transmembrane region (32–54) in phage AP1 HolAP with the N-terminus located outside the membrane, and the C-terminal located inside the membrane, displaying the structural characteristics of type III holin. However, it is opposite to the N-terminal and C-terminal distribution of the typical type III holin in *E. coli* T4 phage [35].

The predicted size of HolAP is about 11.9 kDa, while the size of HolAP is about 17 kDa in Western-blotting analysis. Similarly, the predicted size of LysAP_ΔTMD_ is about 22 kDa, while the size of the extracted LysAP_ΔTMD_ is about 25 kDa. In agreement with the result of this study, the phenomenon has also been reported in some previous studies [2,36]. The difference between theoretical and actual protein size may be main due to both the high isoelectric point and His tag fusion, which have a great influence on the migration of the proteins in SDS PAGE gels.

Because there is only one transmembrane region, it is difficult for holin to form macropores in the cell membrane to release the synthesized endolysin [37]. In this study, β-galactosidase activity assay showed that HolAP could not form macropores for the intracellularly synthesized β-galactosidase and other macromolecular substances to be transported out of the cell membrane. In addition, while TEM showed that the permeability of the cell membrane had been changed, the growth of the bacteria was not inhibited. Related studies have shown that the accumulation of these holins can change in the proton dynamic potential across the cell membrane at a precise time point, resulting in the activation of the inactive endolysin anchored in the cell membrane. The activated endolysin is then released into the periplasm to cleave the cell wall [30].

As previously mentioned, the TMD in LysAP is found in the C-terminal part of the protein, which differs from the traditional endolysin topology (N-terminal) in phages infecting Gram-negative bacteria. Therefore, this study explored the function of the C-terminal TMD by constructing the TMD deletion mutant LysAP_ΔTMD_. Furthermore, it is noteworthy that it was difficult to obtain and purify LysAP protein after induction of IPTG due to severe cell lysis, which is one of the reasons why we purified LysAP_Δ__TMD_ instead. Indeed, when transmembrane region of LysAP was deleted, a single band was observed in LysAP_ΔTMD_ following protein extraction and purification.

Previous studies have shown that about 25% of endolysins in *E*. *coli* phages that cannot synthesize signal peptide have signal-anchoring release domains instead, and the holins encoded by these phages generally form “pinholes” [38,39]. In this study, we identified the function of LysAP, and found that it could lyse bacteria when expressed alone. Bioinformatics analysis predicted that there is a TMD at the C-terminus of LysAP, suggesting that LysAP might have the function of SAR. LysAP cannot lyse the bacteria without the C-terminal TMD. Our previous attempts found the wild type LysAP protein to be challenging to purify in vitro mainly due to the toxicity of LysAP to the bacterial cell. However, the deletion of the C-terminal TMD resulted in high yields of protein produced. These results indicate that the C-terminal TMD plays an important role in the function of LysAP, facilitating anchoring of LysAP in the cell membrane. Single amino acids mutations of cationic residues near LysAP TMD verified the connection between the charge change and LysAP release. In addition, co-expression of HolAP and LysAP was demonstrated to have a synergistic effect on bacterial lysis. However, the specific mechanism of holin, and how LysAP, a C-terminal TMD endolysin, is anchored in the membrane and reaches the periplasm through the cell membrane, need to be further studied.

## 5. Conclusions

The present study identified and investigated the functions of lysis proteins, holin HolAP, and endolysin LysAP in phage AP1, defining a new binary lysis cassette. LysAP alone can be transported to the periplasm via its C-terminal TMD and Sec system. Additionally, with the interaction of LysAP with HolAP, which acts as a probable pinholin, lysis is more efficient. This study could elucidate the phage–bacteria interaction mechanism and provide insights to the biological control of bacterial pathogens.

## Figures and Tables

**Table 1:** Strains, phage, and plasmids.

	Description	Source or Reference
**Strains**		
RS-2	*Acidovorax oryzae*, pathogen of bacterial brown stripe	Lab collection
DH5α	F-Φ80d lacZΔM15Δ(lacZYA-argF) U169 recA1 endA1, hsdR17(rk-, mk+) phoAsupE44 λ- thi-1 gyrA96 relA, *Escherichia coli*	Vazyme
BL21(DE3)	Host for overexpressing proteins driven by T7 promoter, *Escherichia coli*	Invitrogen
BTH101	Host for overexpressing proteins in bacterial two-hybrid, *Escherichia coli*	Chen et al. [15]
**Phage**		
AP1	phage of *Acidovorax oryzae*	Lab collection
**Plasmids**		
pETDuet-1	Amp^R^; expression vector with HIS label	Promega
pETDuet-HolAP	Amp^R^; recombinant expression vector with HIS label with HolAP	This study
pETDuet-LysAP	Amp^R^; recombinant expression vector with HIS label with LysAP	This study
pETDuet-HolAP-LysAP	Amp^R^; recombinant expression vector with HIS label with HolAP and LysAP	This study
pETDuet-LysAP_Δ__TMD_	Amp^R^; recombinant expression vector with HIS label with LysAP_Δ__TMD_	This study
pET-28a(+)	Kan^R^; expression vector with HIS label	Novagen
pET-28a-HolAP	Kan^R^; recombinant expression vector with HIS label with HolAP	This study
pET-28a-LysAP	Kan^R^; recombinant expression vector with HIS label with LysAP	This study
pET-28a-LysAP_Δ__TMD_	KanR; recombinant expression vector with HIS label with LysAP_Δ__TMD_	This study
pET-28a-LysR222A	Kan^R^; point mutation recombinant expression vector with HIS label with LysAP	This study
pET-28a-LysE15A	Kan^R^; point mutation recombinant expression vector with HIS label with LysAP	This study
pKNT25	Kan^R^; expression vector for bacterial two-hybrid test	Chen et al. [15]
pKNT-LysAP	Kan^R^; recombinant expression vector for B2H test	This study
pCH363	Amp^R^; expression vector for bacterial two-hybrid test	Chen et al. [15]
pCH-HolAP	Amp^R^; recombinant expression vector for B2H test	This study

Kan^R^, Amp^R^, indicate Kanamycin-, Ampicillin-resistant, respectively.

**Table 2:** Primers used in this study.

Primers Name	Sequences (5′-3′)	Length
pETDuet-LysF	CTATACATATGATGAAAACCTCTGATCGCGGAC	684 bp
pETDuet-LysR	GAAGATCTTGACCACCCCTCTCGCCG	
pET28a-LysF	CGGGATCCATGAAAACCTCTGATCGCGGAC	684 bp
pET28a-LysR	CCCAAGCTTTGACCACCCCTCTCGCCG	
Lys_△__TMD_F	CGGGATCCATGAAAACCTCTGATCGCGGACTCGC	615 bp
Lys_△__TMD_R	CCCAAGCTTTGACCACCCCTCTCGCCGCACCTTCACACGCTCGTCAGCGCTCGACTTGATG	
E15A-F	AGCAAATGGGTCGCGGATCCATGAAAACCTCTGATCGCGGACTCGCGCTGATCGAAGAATTCGCGGGCTTC	684 bp
E15A-R	TCGAGTGCGGCCGCAAGCTTTCATGACCACCCCTCTCGCC	
R222A-F	AGCAAATGGGTCGCGGATCCATGAAAACCTCTGATCGCGGACTCGC	684 bp
R222A-R	TCGAGTGCGGCCGCAAGCTTTCATGACCACCCCTCTGCCCGCACCTTCACACG	
pETDuet-HolF	TATGCCATGGATGCAATCCATGAATGTCGAAAC	336 bp
pETDuet-HolR	CGGGATCCCTTAGCAGACTCGAGTGCG	
pET28a-HolF	CGGGATCCATGCAATCCATGAATGTCGAAACC	336 bp
pET28a-HolR	CCCAAGCTTCTTAGCAGACTCGAGTGCG	
pCH-HolF	GCAAGCTTATGCAATCCATGAATGTCGAAACC	336 bp
pCH-HolR	CGGAATTCCTTAGCAGACTCGAGTGCG	
pKNT-LysF	CGGGATCCATGAAAACCTCTGATCGCGGAC	684 bp
pKNT-LysR	TATAGAATTCTGACCACCCCTCTCGCCG	

Note: Nucleotides with underline indicated restriction sites of the enzymes: BamHI, EcoRI, NdeI, BglII, and NcoI.

## Data Availability

All data supporting the conclusions of this article are included in this article. The genome sequences of AP1 have been deposited at GenBank database with Accession No. OM049504.
